# Cardiac Baroreflex Variability and Resetting during Sustained Mild Effort

**DOI:** 10.3389/fphys.2017.00246

**Published:** 2017-05-05

**Authors:** Mair Zamir, Mark B. Badrov, T. Dylan Olver, J. Kevin Shoemaker

**Affiliations:** ^1^Department of Applied Mathematics, Western UniversityLondon, ON, Canada; ^2^Department of Medical Biophysics, Western UniversityLondon, ON, Canada; ^3^School of Kinesiology, Western UniversityLondon, ON, Canada; ^4^Department of Physiology and Pharmacology, Western UniversityLondon, ON, Canada

**Keywords:** baroreflex function, baroreflex resetting, baroreflex variability, logistic curve, spontaneous sequence analysis

## Abstract

This exploratory study assessed the pattern of closed-loop baroreflex resetting using multi-logistic-curve analysis. Operating point gain and ranges of RR-interval (RRI) and systolic blood pressure (SBP) are derived to examine how these relate to sympathetic activation. Sustained low-intensity isometric handgrip exercise, with a period of post-exercise circulatory occlusion (PECO), provided a model to study baroreflex resetting because the progression toward fatigue at constant tension induces a continuous increase in volitional contribution to neuro-cardiovascular control. Continuous measurements of muscle sympathetic nerve activity (MSNA), blood pressure, and RRI were made simultaneously throughout the experimental session. Spontaneous sequence analysis was used to detect episodes of baroreflex “engagements”, but the results are examined with a view to the fundamental difference between experimental conditions that isolate the carotid sinus (open-loop) and intact physiological conditions (closed-loop). While baroreflex function under open-loop conditions can be described in terms of a single logistic curve, intact physiologic conditions require a *family of logistic curves*. The results suggest that the baroreflex is in a “floating” state whereby it is continuously resetting during the timeline of the experiment but with minute-by-minute average values that mimic the less complex step-wise resetting pattern reported under open-loop conditions. Furthermore, the results indicate that baroreflex function and resetting of the operating point gain is reflected not in terms of change in the *values* of blood pressure or RR-interval but in terms of change in the *range of values* of these variables prevailing under different experimental conditions.

## Introduction

Baroreflex control over heart rate is commonly described by a logistic curve relating systolic blood pressure (SBP) and RR-interval (RRI) or heart rate (HR). The logistic curve provides a point of maximum gain (MG) and points of operating gain (OG), the latter reflecting the physiologic state at the time of measurement. Measured under open-loop conditions, such as in the neck cuff model, the SBP-HR curve has been found to reset upward and to the right during progressive volitional exercise, with little change in maximal gain, but with progressive decline in operating gain as heart rate increases (Rowell and O'Leary, 1990; Rowell, 1993; Raven et al., 2006). While the neural pathways involved remain unclear, the general view regarding the resetting process during volitional exercise involves both top-down neural input (central command; Raven et al., 2002) as well as bottom-up inputs (muscle metaboreflex; Alam and Smirk, 1937; McCloskey and Mitchell, 1972; Kaufman et al., 1983; Mitchell et al., 1983; Potts and Mitchell, 1998).

Some key questions remain regarding the concept of baroreflex resetting in the context of top-down or muscle reflex inputs. For example, the logistic nature of the baroreflex curve defines not only the maximal and the operating gains but also the operating point of the reflex as well as ranges of SBP and RRI, features that often are ignored. Other determining factors include the nature of the exercise stimulus itself. During dynamic (e.g., cycling) exercise, open-loop models of baroreflex function suggest that the range of RRI and SBP narrow as the intensity of dynamic exercise increases (see Raven et al., 2006 for review). However, with isolated metaboreflex activation these single logistic curves retain their range and the curve shifts rightward to higher blood pressures without changes to maximal gain.

This view of baroreflex resetting is contingent upon the existence of a single SBP-RRI logistic curve which can be used as a reference “baseline state” of the baroreflex and with which other states can be compared. However, data obtained under *intact physiological (closed-loop) conditions* indicate that baroreflex function produces highly scattered SBP-RRI data at baseline which cannot be described in terms of a single logistic curve (Zamir et al., 2014). A single logistic curve describing baroreflex function can be found only under *open-loop conditions* such as those produced under experimental conditions that isolate a baroreceptor population and/or inhibit the vasomotor response (Chen and Bishop, 1983; Barbieri et al., 2001). Thus, under intact physiological conditions a single point of maximum gain does not exist (Schwartz et al., 2013), and it is likely that the *ranges of SBP and RRI* are important operating variables in this case because these variables combine to describe the operating “mode” of the reflex.

Currently, open-loop models provide the basis of our understanding regarding baroreflex resetting during exercise, but the fundamental difference between open-loop and closed-loop models of baroreflex control of heart rate cannot be ignored. The limitations and challenges associated with each model create confusion and are often debated (Parati et al., 2000; Lipman et al., 2003; Parati, 2005; Diaz and Taylor, 2006; Zamir et al., 2014). Of concern in the current study is the fact that open loop conditions produce a single logistic curve to describe the dynamics of the baroreflex, which confines the SBP-RRI relationship and hence the scope of baroreflex dynamics to that specific curve (Kent et al., 1972). A key feature of a single SBP-RRI logistic curve is the point at which the baroreflex operates at MG; this gain then declines at other points along the curve as SBP and RRI increase or decrease, that is, as the operating point moves away from the maximum gain point. Baroreflex “resetting” in this context has been described in terms of either a shift in the position of the baroreflex SBP-RRI operating point along one logistic curve, a shift in the logistic curve itself, or both (Raven et al., 2006).

In the present, essentially exploratory, study we take the view that the scattered SBP-RRI data describing baroreflex function under intact physiological conditions define not a single logistic curve but a *family of logistic curves* (Zamir et al., 2014) that, in turn, define an SBP-RRI *space* within which the baroreflex is operating. This *operational space* of the baroreflex, which is determined directly by the measured SBP-RRI data, provides a measure of the *ranges* of SBP and RRI (or heart rate) prevailing during baroreflex function under closed-loop physiological conditions. The range and variability of heart rate have been used extensively as a diagnostic utility in relation to a number of pathologic conditions and trauma (Kleiger et al., 1987; Barron and Viskin, 1998; Cohen et al., 1998; Abildstrom et al., 2003; Nickel and Nachreiner, 2003; Shaffer et al., 2014), here we explore the extent to which these relate to baroreflex function.

Isometric handgrip exercise with sustained mild effort offers a unique experimental model to study baroreflex resetting because the progression toward fatigue at constant tension induces a continuous increase in volitional contribution to neuro-cardiovascular control. Then, following the cessation of effort, a period of post-exercise circulatory occlusion (PECO) provides the opportunity to observe sustained metaboreflex contributions to neuro-cardiovascular control in the absence of central command (Alam and Smirk, 1937). The metaboreflex, triggered by fatigued muscle in the absence of volitional effort, reportedly has little effect on the SBP-RRI operating point, compared to baseline, owing to heart rates that return to baseline levels but at a higher blood pressure (Gallagher et al., 2001). Accordingly, in the present study we examine the patterns of change in SBP-RRI sequences, *as well as the prevailing ranges of SBP and RRI*, during fatiguing exercise followed by a period of PECO to quantify metaboreflex contributions independent of volitional effort (Alam and Smirk, 1937). The main purpose of the study is to explore the nature of baroreflex resetting under intact physiological conditions.

## Methods

### Participants

Five healthy male volunteers (27 ± 4 years; 178 ± cm; 83 ± kg) participated in the study after providing written informed consent, and following at least a 3-h fast, a 12-h abstinence from caffeine, alcohol, and other stimulants, and a 24-h abstinence from vigorous exercise. Participants were recreationally active, non-smokers, and non-medicated, with no history of overt cardiovascular or respiratory disease. All study protocols were approved by the Health Sciences Research Ethics Board at Western University, Canada.

### Experimental protocol

All testing was conducted with participants in the supine position. Following a 3-min baseline rest period, participants performed 5 min of isometric handgrip (HG) exercise with their non-dominant hand at 20% of their pre-determined maximal voluntary contraction (MVC). Approximately 5 s prior to exercise completion, a pneumatic cuff (Hokanson SC12D, D.E. Hokanson, Inc., Bellevue, Washington, USA) on the exercised upper arm was inflated via a rapid cuff inflator (Hokanson E20 Cuff Inflator, D.E. Hokanson, Inc., Bellevue, Washington, USA) to a pressure above SBP (~200 mmHg) to initiate a period of PECO for 4 min. Subsequently, the cuff was deflated and a 3-min recovery period was initiated. Participants were asked to rate their level of perceived exertion following exercise completion using the Borg Rating of Perceived Exertion Scale (Borg, 1970). The reported rates ranged from 14/20 to 18/20.

### Experimental measures

**Figure 1:**
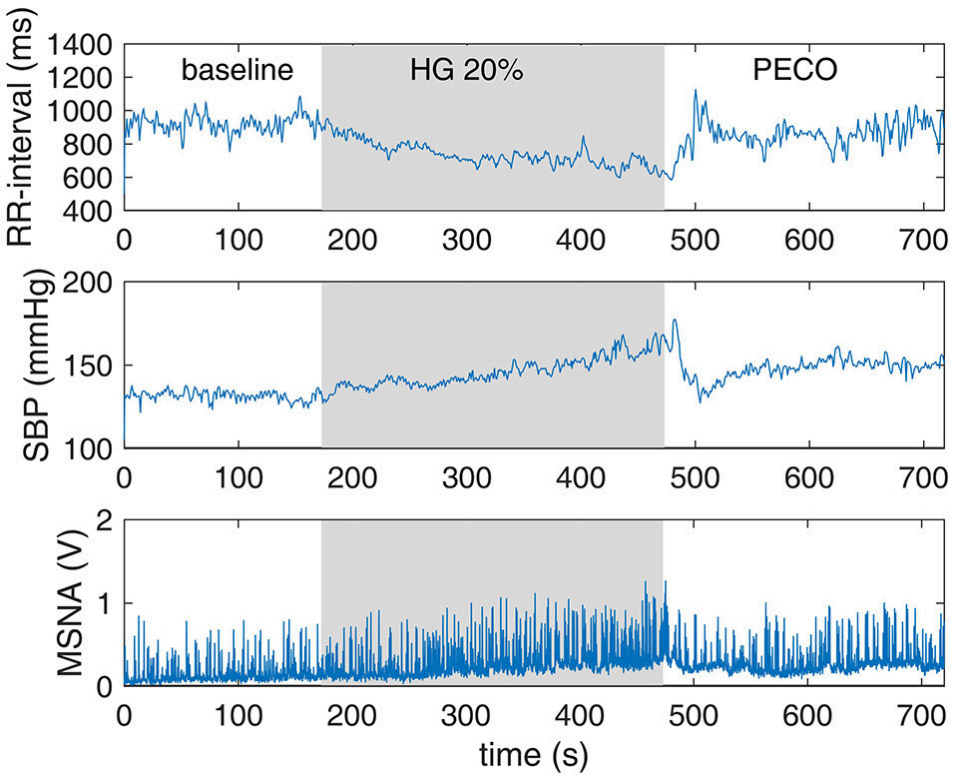
**Continuous measurements of RR-interval, systolic blood pressure (SBP), and muscle sympathetic nerve activity (MSNA) made simultaneously during the three phases of the experiment**.

Continuous measurements of muscle sympathetic nerve activity (MSNA), blood pressure, and heart rate were made throughout the protocol. Sympathetic nerve recordings were obtained in the right peroneal nerve by microneurography (Hagbarth and Vallbo, 1968), using methods from our laboratory described previously in detail (Kimmerly and Shoemaker, 2003; Kimmerly et al., 2004; Badrov et al., 2015; Usselman et al., 2015). Beat-to-beat blood pressure was measured using finger photoplethysmography (Finometer; Finapres Medical Systems, Amsterdam, The Netherlands). Blood pressure values obtained from the Finometer were calibrated to the average of three baseline blood pressure measurements assessed using manual sphygmomanometry. Heart rate was measured throughout using a standard three-lead electrocardiogram. All data were collected and analyzed offline using PowerLab/16SP with LabChart 6 (ADInstruments, Colorado Springs, Colorado, USA). Typical measurements from one subject are shown in Figure 1.

### Data analysis

**Figure 2:**
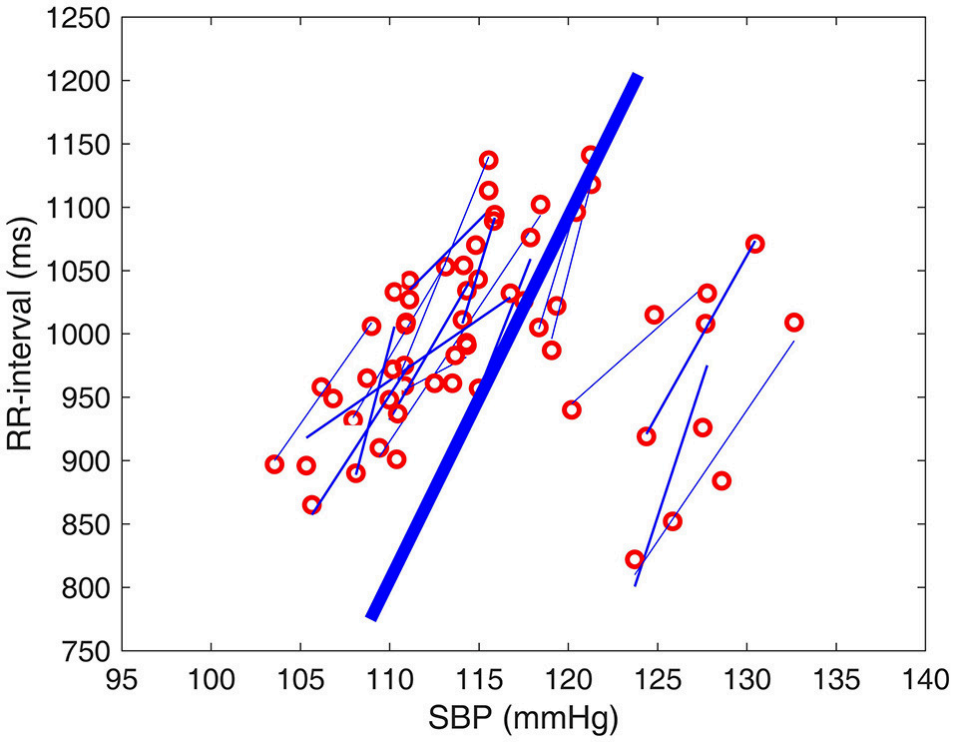
**Episodes of baroreflex engagements as normally presented in spontaneous sequence analysis, based on data from one subject at baseline**. An episode consists of three or more consecutive cardiac cycles (red circles) during which SBP and RRI both increase or decrease from the first cycle to the second and from the second to the third etc. The episode thus provides three or more data points from which to extract a regression line (thin blue lines). The slope of each of these lines is considered to be a measure of the “gain” or “sensitivity” of the baroreflex during that engagement episode, and the average of all such slopes (thick blue line) is considered to be a measure of the average sensitivity of the baroreflex during that particular experimental session.

Spontaneous sequence analysis was used as described previously (Bertinieri, 1985; Blaber et al., 1995; Parati et al., 2000; Moffitt et al., 2005; Stauss et al., 2006; Laude et al., 2008; Hollow et al., 2011) to determine episodes of baroreflex “engagement” during the experimental session. Briefly, an engagement episode involves 3 or more consecutive cardiac cycles during which SBP and RRI both increase or decrease from the first cycle to the second and from the second to the third, etc. The episode thus provides three (or more) data points in the SBP-RRI plane from which to extract a regression line. Typical results from one subject are shown in Figure 2.

The use of multi-logistic curves to examine baroreflex function has been described previously (Zamir et al., 2014). Briefly, SBP-HR data, which are highly scattered under normal closed-loop operation, are used to define a mean logistic curve based on the mean values of SBP and HR, writing

$$\begin{array}{l} y\left(x\right)=b+c\times \tanh\left(d\times \left(x-a\right)\right) \end{array}$$

where *x, y* represent SBP and RRI data, respectively, *a, b* are their corresponding mean values, *c* × *d* is mean slope (sensitivity), and “tanh” is the hyperbolic tangent function.

$$\begin{array}{l} \tanh x=\frac{e^{2x}-1}{e^{2x}+1} \end{array}$$

Two other logistic curves on either side are then obtained by extending the values of the parameters *a, b, c, d* by ±0.5 and ±1.0 standard deviations as determined from the data. The result is a family of logistic curves whose properties and relative disposition reflect the scatter of the SBP-RRI data as shown in **Figure 4**. The immediate advantage of this picture is that it shows to what extent the baroreflex is operating along a single logistic curve (as in an open-loop setting) and to what extent it is deviating from this construct. In the present study we used this scheme to examine the dynamic time-varying behavior of the baroreflex, particularly as it relates to the concept of resetting, during progressively fatiguing handgrip exercise that raises blood pressure and heart rate.

#### Statistics

A repeated measures two-way analysis of variance (ANOVA) assessed the effect of protocol time with levels of significance as indicated in the figures and captions (**Figures 5**–**8**). It is important to emphasize that the main purpose of this analysis was not to examine variance between subjects but rather to determine the level of significance of variance between the state of baroreflex at baseline and at different stages of the HG session.

## Results

**Figure 3:**
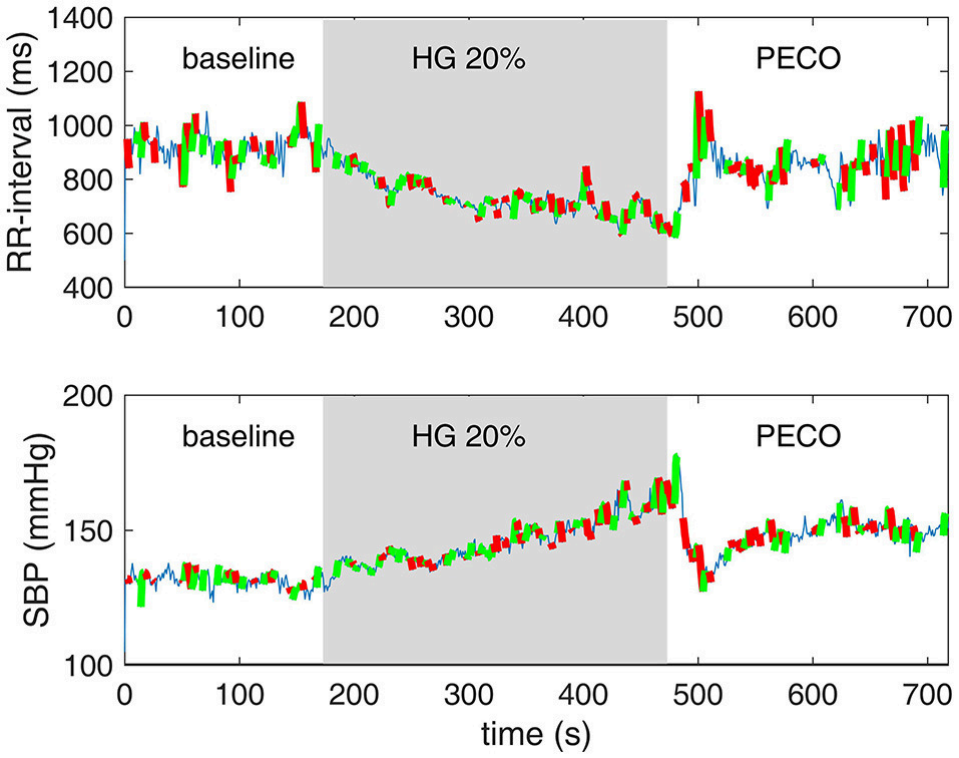
**Episodes of baroreflex engagements as normally presented in spontaneous sequence analysis (Figure **2**) are here placed along the timeline of the experiment**. Episodes in which SBP and RRI were increasing in tandem are shown in green, and those in which SBP and RRI we decreasing in tandem are shown in red. Thin blue lines indicate periods in which the baroreflex was not engaged.

Typical measurements from one subject for the time course of RRI, SBP, and MSNA are shown in Figure 1. We extended the utility of these measurements by placing the engagement episodes along the time-line of the experiment, and by distinguishing between episodes of increasing or decreasing SBP and RRI, as shown in Figure 3.

**Figure 4:**
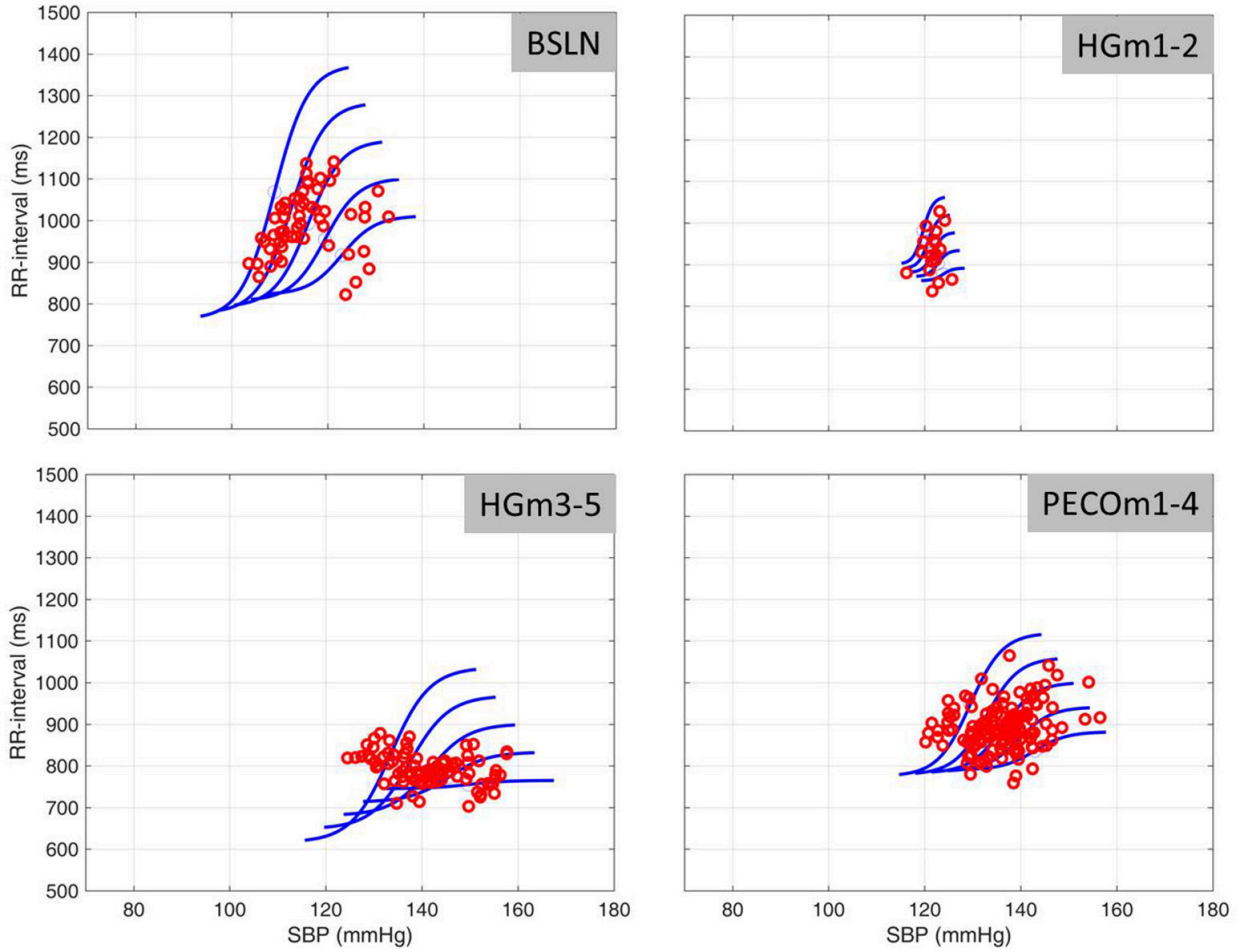
**SBP-RRI data as determined by spontaneous sequence analysis from one subject**. The dots refer to the spontaneous operating points of the SBP-RRI data in each of the Baseline (BLN), first 2 min of handgrip exercise (HBm1–2), minutes 3–5 of handgrip (HGm3–5) and during a 4 min period of post-exercise circulatory occlusion (PECO). Logistic curves represent the mean (middle curve of each group) plus or minus two standard deviations (outside curves to the right or left of the center average curve. The position and slopes of the baroreflex “space” shift to the right and downward as exercise progresses, recovering somewhat during PECO.

**Figure 5:**
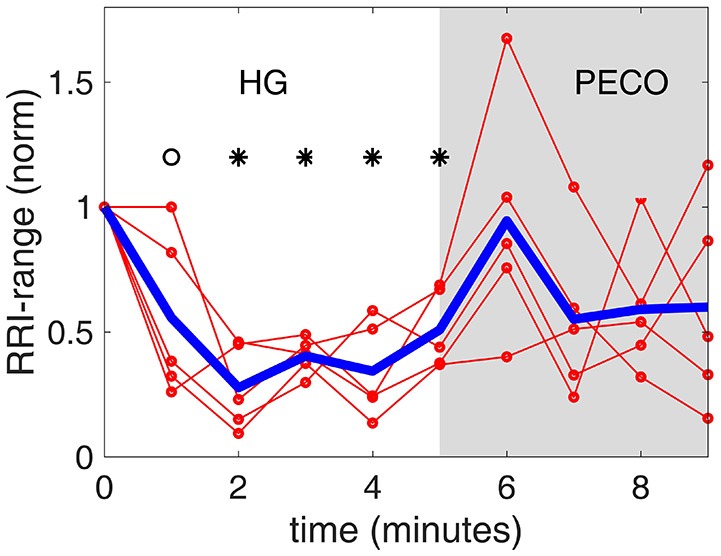
**The ***range*** of RR interval values [RRI-range, normalized to a value of 1.0 at baseline (time “0”)] observed at each minute during the handgrip exercise (HG) and post-exercise circulatory occlusion (PECO)**. Specifically, while RRI is seen to decline monotonically during the 5 min HG in Figure 1, RRI-range is seen here to drop dramatically in the first 2 min of HG then stabilizing and rising again. The thin red lines representing individual subjects are shown to illustrate the differences that exist among subjects, while the heavy blue line represents the average of all subjects. 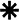
Significantly different from baseline (*p* < 0.01). 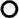
Significantly different from baseline (*p* < 0.05).

**Figure 6:**
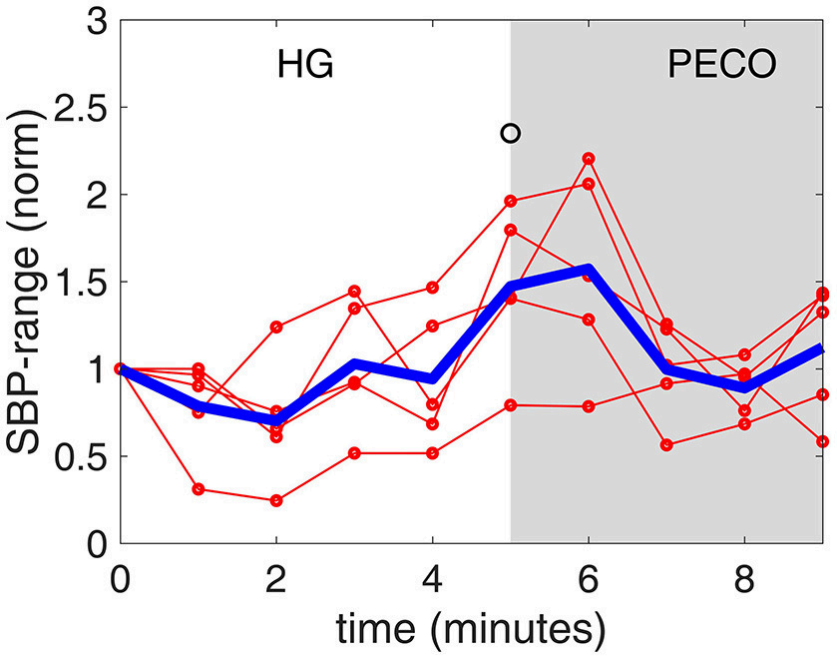
**The ***range*** of systolic blood pressure values [SBP-range, normalized to a value of 1.0 at baseline (time “0”)] observed at each minute during the handgrip exercise (HG) and post-exercise circulatory occlusion (PECO)**. Again there is a drop in SBP-range in the first 2 min, then a rise to above pre-HG level in the next 3 min. By contrast, SBP itself rises monotonically during the 5 min of HG, as seen in Figure 1. The thin red lines representing individual subjects are shown to illustrate the differences that exist among subjects, while the heavy blue line represents the average of all subjects. 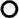
Significantly different from baseline (*p* < 0.05).

The range of SBP-RRI sequences measured at baseline for a single individual is shown in the first panel of Figure 4. Overall, the results indicate that the rise in heart rate and blood pressure during the 5 min of handgrip was one-directional and fairly continuous as seen in Figures 1, 3 (not to be confused with the increasing-decreasing RRI and SBP occurring on the much smaller scale of baroreflex engagements referred to above). By contrast, the *range* of heart rate and blood pressure within which the changes occurred, as measured by their standard deviations, showed a significantly different pattern. Specifically, Figure 5 shows that there is an immediate drop in the range of RRI at the onset of the HG session, persisting for 2 min or so, then rising gradually in the next 3–4 min and stabilizing toward the end of PECO. The corresponding changes in the range of SBP were more moderate and statistically insignificant as shown in Figure 6.

**Figure 7:**
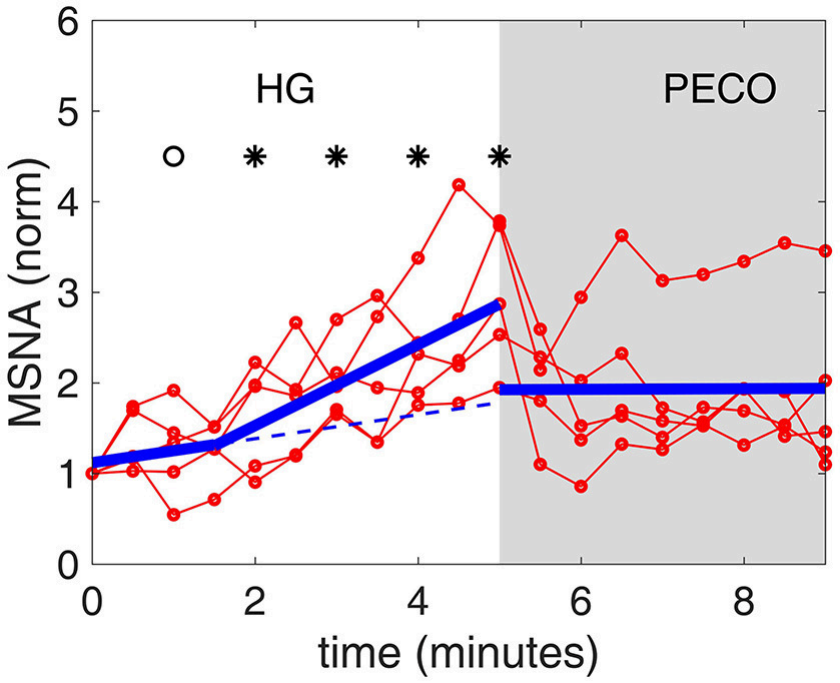
**Muscle sympathetic nerve activity [MSNA, normalized to a value of 1.0 at baseline (time “0”)] during the timeline of the experiment**. There is a distinct change in the rate of increase following the first 2 min or so of HG. The thin red lines representing individual subjects are shown to illustrate the differences that exist among subjects, while the heavy blue line represents the average of all subjects. The dashed blue line represents a continuation of the slope during the first 2 min, to highlight the change in slope during the next 3 min. 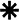
Significantly different from baseline (*p* < 0.01). 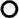
Significantly different from baseline (*p* < 0.05).

Figure 7 illustrates the change in MSNA during the experimental session. As expected (Badrov et al., 2016), the general trend was a continuous increase in MSNA during the 5 min of HG, that stabilized during the 4 min of PECO. However, a distinct difference was observed in the *rate of increase*, being slow during the first 2 min or so of HG compared with a rapid increase during the final 3 min of HG.

**Figure 8:**
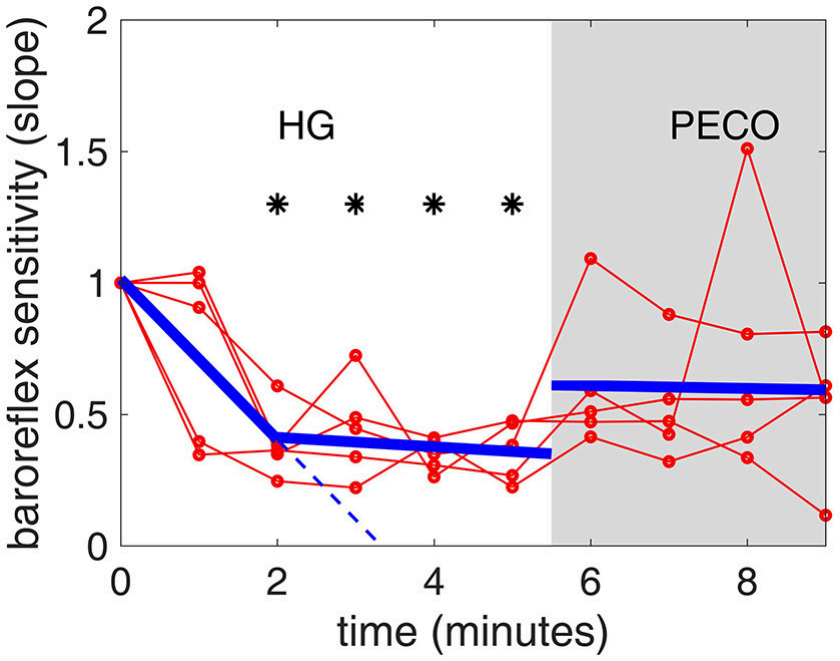
**Baroreflex sensitivity during the timeline of the handgrip (HG) and post-exercise circulatory occlusion (PECO) phases of the experiment [nornmalized to a value of 1.0 at baseline (time “0”)]**. There is a steep drop in sensitivity during the first 2 min of HG, followed by a less steep but continuing drop in the next 3 min. The thin red lines representing individual subjects are shown to illustrate the differences that exist among subjects, while the heavy blue line represents the average of all subjects. The dashed blue line represents a continuation of the slope during the first 2 min, to highlight the change in slope during the next 3 min. 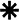
Significantly different from baseline (*p* < 0.01).

Figure 8 illustrates the time course of changes in baroreflex sensitivity during the 5 min of HG and 4 min of PECO, as measured by the slopes of RRI-SBP sequences in Figure 2, normalized to baseline. Both the individual and the averaged responses are illustrated in this figure. The general trend was a rapid decline in baroreflex sensitivity during the first 2 min of HG with a relatively stable level thereafter, followed by stabilization at a new level (between baseline and end-HG levels) during the 4 min of PECO. While the figure shows minute-by-minute averages, we also provide a video of *real-time* shifts in the baroreflex sequences throughout the experimental protocol (see Supplemental Material).

## Discussion and conclusions

A measure of baroreflex operating point “sensitivity” can be determined by the SBP-RRI slopes obtained from spontaneous sequence analysis (Blaber et al., 1995; Parati et al., 2000; Persson et al., 2001; Parati, 2005). We used this methodology to provide, for the first time, a demonstration of the *dynamic* pattern of change in spontaneous cardiac baroreflex function during isometric handgrip effort and metaboreflex activation.

The results indicate a “floating” baroreflex operating point at rest, a rapid downward resetting of baroreflex sensitivity (slope) in the first 2 min of HG, followed by a stable level of sensitivity over the final 3 min of HG. This pattern of cardiac baroreflex change occurs coincidentally with the increase in MSNA during the final 3 min HG seen in Figure 7. The PECO phase produced an incomplete recovery of baroreflex sensitivity and ranges of RRI and SBP, despite normalization of heart rate to baseline levels, indicating that both top-down and metaboreflex processes were contributing to the resetting pattern during exercise. Some of these patterns have in the past been viewed, using the open-loop concept, as “resetting” of the baroreflex in terms of a change in its operating position along a representative RRI-SBP logistic curve or in terms of a shift in the position of the logistic curve itself within the RRI-SBP plane. Our results indicate that baroreflex function under closed-loop conditions involves dynamic changes in both the operating point slope and the range of RRI within the baroreflex “space”, thus exposing these as important variables in understanding baroreflex responses.

In its common use, the spontaneous sequence analysis method for the assessment of baroreflex function (Parati et al., 2000; Parati, 2005; Pinna et al., 2015) relies on *averaging* the measured sensitivities and thus missing the dynamic nature of the reflex, and ignoring changes in the range of values of the variables contributing to the change in average slope. Also, the method may capture non-specific SBP-RRI associations (Diaz and Taylor, 2006) and randomly formed positive and negative sequences that can be interpreted as baroreflex sensitivity curves (Blaber et al., 1995). However, pharmacological evidence has shown that muscarinic blockade minimizes the slopes of sequence segments (Zamir et al., 2014), indicating that the majority of the sequences obtained under normal conditions are in fact driven by cardiovagal pathways.

Resolution of the time-course of changes in baroreflex sensitivity provides a new dimension in the study of baroreflex physiology. For example, when the baroreflex is not interrupted by external interventions, the SBP-RRI sequences observed are not stable at rest (Zamir et al., 2014), being characterized by a range of similar (but not identical) slopes. These observations reflect a “floating” baroreflex operating point with a fairly wide range of sensitivities at baseline but a narrower range during exercise. A demonstration of this in real time is provided in the Supplemental Video. Even with minute-by-minute averaging, however, the current study indicated that baroreflex sensitivity decreased rapidly within the first 2 min of exercise with a corresponding reduction in range of RRI, suggesting a focusing of the baroreflex around a new operating point.

Collectively, these findings suggest the intriguing notion that the behavior of the baroreflex is reflected not in terms of a step change in the average value of its sensitivity from one set of experimental conditions to the next but in terms of continuous change along the timeline of the experiment. The real-time behavior, as shown in the Supplementary Video, suggests an unstable operating point at each stage, a behavior that can challenge interpretations based on averaged values or open-loop modeling.

**Figure 9:**
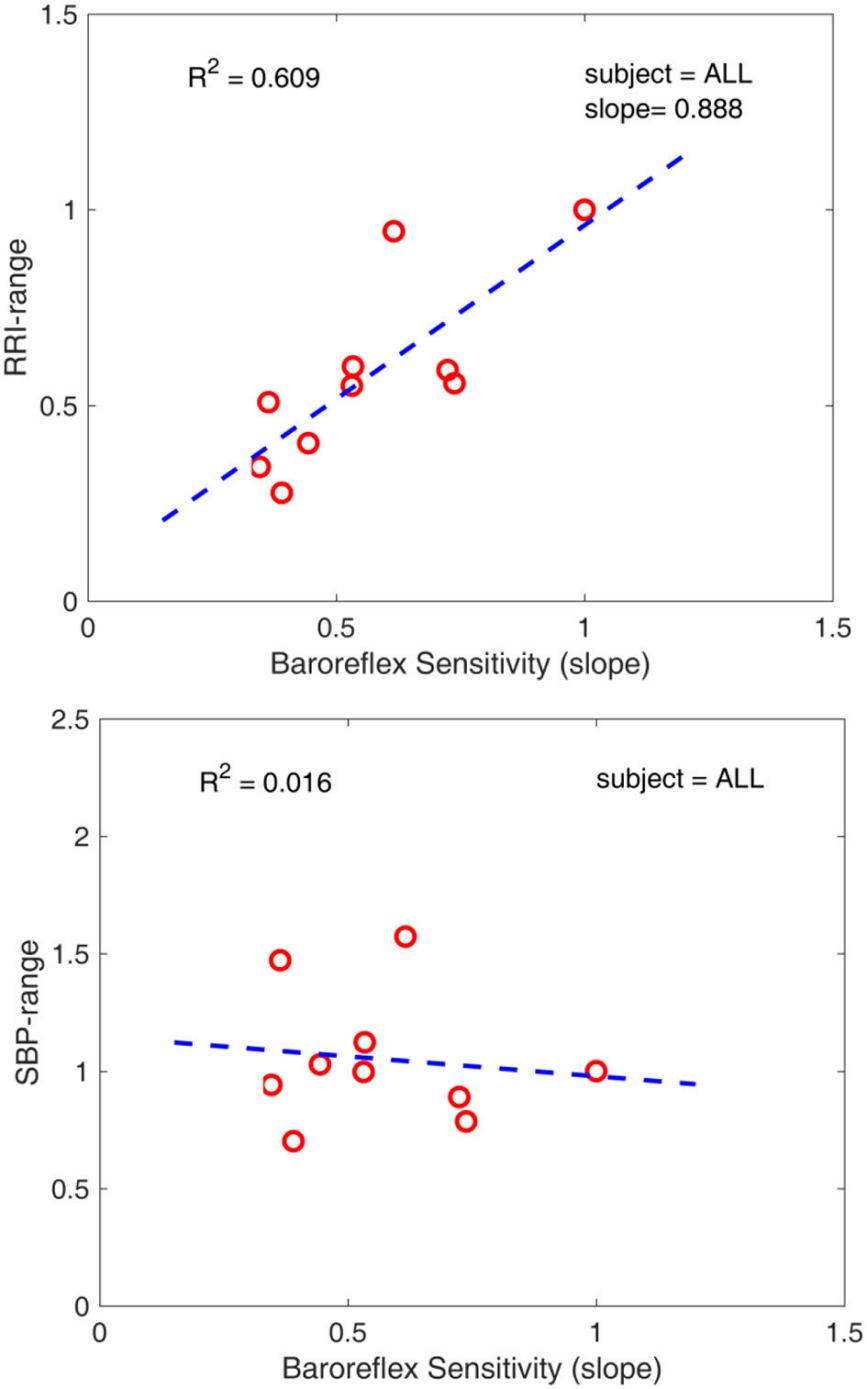
**(top)** Correlation between baroreflex sensitivity and RRI-range. Low RRI-range is associated with low baroreflex sensitivity. **(bottom)** Lack of correlation between baroreflex sensitivity and SBP-range. The 10 data points in each panel represent the 10 data points of average of the 5 subjects in each of Figures 5,6,8.

The existence of two distinct phases in the *ranges* of RRI and SBP (Figures 5, 6) in tandem with the two phases of change in baroreflex sensitivity indicates that the range of values of RRI and/or SBP become important markers of, or contributors to, baroreflex activity. This is supported by the fact that the sharp drop in baroreflex sensitivity at the onset of exercise is accompanied by a sharp drop in RRI-range. Indeed, the two drops are well correlated as shown in Figure 9, top panel: the range of RRI is higher when the baroreflex is operating at higher sensitivity (gain). This pattern reinforces observations of stable maximal gain, reduced operating point, and reduced RRI-range, made by the neck-cuff model of cardiac baroreflex function during exercise (Fisher et al., 2013). By contrast, the results in Figure 9, bottom panel, indicate that there is no correlation between baroreflex sensitivity and SBP-range.

The picture that emerges from these findings is that during the first 2 min of HG the rise in blood pressure is mediated largely by volitional mechanisms as indicated by the large drop in the range of values of RRI and the very small increase in the level of MSNA. However, a distinct change in the directions of baroreflex operating gain and RRI range occurs at this point of sympathetic activation (3 min of the HG phase), likely reflecting the onset of metaboreflex from the active muscle.

The differential effect of metaboreflex vs. “central” influences on cardiac baroreflex function have been studied in some detail in the past, resulting in a focus on variations in parasympathetic contributions to heart rate control. For example, on the basis of observations in exercising dogs under conditions of graded vascular occlusion with cardiac autonomic blockade O'Leary (1991, 1996) argued that a surge of parasympathetic outflow occurs during metaboreflex activation that dominates concurrent sympathetic cardiac activation, thereby reducing heart rate while maintaining high baroreflex gain. Functionally, this dominant vagal effect produces a heart rate that returns to baseline levels despite very high levels of sympathetic drive. Recently, Fisher et al. (2013) used a neck-cuff model of carotid sinus stimulation under conditions of cardiac autonomic blockade in humans and argued that sympathetic activation forms a primary determinant of the heart rate response to fatiguing exercise and that contributions from vagal withdrawal occur only in larger muscle mass conditions. Using spontaneous sequence methods, such as those used in the current study, and a model of graded partial flow restriction in humans, followed by a period of complete ischemia, Hartwich et al. (2011) reported that the metaboreflex diminished cardiac baroreflex function only in leg exercise, not in forearm exercise. In fact, they did not observe any change in cardiac baroreflex function in any of the free-flow, partial flow restriction, or complete flow restriction and PECO components of the handgrip protocol. These earlier studies contrast with the present study, possibly because of different exercise models (rhythmic handgrip in the earlier study and isometric handgrip in the current study). Nevertheless, the conclusion that metaboreflex activation *per se* in handgrip exercise fails to produce marked changes in cardiac baroreflex function continues to hold in open-loop (Raven et al., 2006; Fisher et al., 2008), closed-loop (Hartwich et al., 2011), as well as in the present study. However, the time-course model used in the present study raises the interesting speculation that the metaboreflex may interfere with central command-based downward shifting of the RRI-SBP reflex curve.

The mechanisms underlying cardiac baroreflex resetting are not fully understood at present. Some existing evidence points strongly to a dominant role for parasympathetic involvement. For example, β-1 cardiac blockade with metoprolol did not modify the cardiac baroreflex resetting pattern during dynamic cycling exercise as assessed with the neck suction/pressure method (Ogoh et al., 2005). Yet, more recent outcomes from neck-suction/pressure studies during incremental exercise with cardiac blockade (White and Raven, 2014), suggest that vagal mechanisms dominate resetting early in exercise, but diminish as the exercise intensity increases. An interpretation of these previous findings, together with the current findings that sympathetic drive remains strong during PECO while BRS and SBP ranges return toward baseline levels during PECO, is that sympathetic activation during fatiguing handgrip exercise has little direct effect on the pattern of cardiac baroreflex resetting observed in the present study.

In summary, our results lead to the novel concept that under baseline conditions the cardiac baroreflex is in a “floating” state whereby its function is variable and unstable and operates within a family of logistic curves in an SBP-RRI “space.” During arousal, baroreflex function is more “focused” with a tightening of both the RRI range and the overall operating point gain. The results suggest further that metaboreflex activation modifies the trajectory of both the operating point gain and the RRI range induced by volitional activity.

In the way of limitations, our study was restricted to baroreflex function under sustained mild effort for the stated reasons. Our conclusions, therefore, carry the same restrictions. Baroreflex behavior at high and maximal effort remains to be explored. The number of participants was small, although it was used merely to illustrate qualitative behavior rather than as a statistical sample. In addition, ventilation is known to exert some impact on SBP–RR interval sequences (Hollow et al., 2011). This effect was not measured or controlled in the current study although Valsalva's were not permitted. However, the patterns of BRS are consistent with those reported by users of the neck-cuff model where cessation of ventilation is required to make the measurements.

## Author contributions

MZ, MB, TO, and JS, contributed to all aspects of the study.

### Conflict of interest statement

The authors declare that the research was conducted in the absence of any commercial or financial relationships that could be construed as a potential conflict of interest.
